# Association of MYH9-rs3752462 polymorphisms with chronic kidney disease among clinically diagnosed hypertensive patients: a case-control study in a Ghanaian population

**DOI:** 10.1186/s40885-020-00148-w

**Published:** 2020-08-01

**Authors:** William K. B. A. Owiredu, Michael Appiah, Christian Obirikorang, Evans Asamoah Adu, Vincent Boima, Ernestine Kubi Amos-Abanyie, Priscilla Abena Akyaw, Eddie-Williams Owiredu, Emmanuel Acheampong

**Affiliations:** 1 Department of Molecular Medicine, School of Medicine and Dentistry, Kwame Nkrumah University of Science and Technology, Kumasi, Ghana; 2 Department of Medicine and Therapeutics, School of Medicine and Dentistry, College of Health Sciences, University of Ghana, Accra, Ghana; 3 H3Africa Kidney Disease Research Project, Noguchi Memorial Institute for Medical Research, University of Ghana, Accra, Ghana; 4 School of Medical and Health Science, Edith Cowan University, Joondalup, Australia

**Keywords:** Chronic kidney disseise, Hypertension, Single nucleotide polymorphism, MYH9- rs3752462

## Abstract

**Background:**

Chronic kidney disease (CKD) is a significant comorbidity among hypertensive patients. Polymorphisms in the non-muscle myosin heavy chain 9 gene (MYH9) have been demonstrated to be significantly associated with CKD, among African- and European-derived populations. We investigated the spectrum of MYH9-associated CKD among Ghanaian hypertensive patients.

**Methods:**

The study constituted a total of 264 hypertensive patients. Hypertensive patients with glomerular filtration rate (eGFR) < 60 ml/min/1.73m^2^ (CKD-EPI formula) or clinically diagnosed were defined as case subjects (*n* = 132) while those with eGFR ≥60 ml/min/1.73m^2^ were classified as control subjects (*n* = 132). Demographic data were obtained with a questionnaire and anthropometric measurements were taken. Five (5) millilitres (ml) of venous blood was drawn from study subjects into gel and EDTA vacutainer tubes. Two (2) mL of EDTA anticoagulated blood was used for genomic DNA extraction while three (3) mL of blood was processed to obtain serum for biochemical measurements. Genotyping of MYH9 polymorphisms (rs3752462) was done employing Tetra primer Amplification Refractory Mutation System (T-ARMS) polymerase chain reaction (PCR). Spot urine samples were also collected for urinalysis. Hardy-Weinberg population was assessed. Logistic regression models were used to assess the associations between single nucleotide polymorphisms and CKD.

**Results:**

The cases and control participants differed in terms of age, sex, family history, and duration of CKD (*p*-value < 0.001). The minor allele frequencies of rs3752462 SNP were 0.820 and 0.567 respectively among the control and case subjects. Patients with the heterozygote genotype of rs3752462 (CT) were more likely to develop CKD [aOR = 7.82 (3.81–16.04)] whereas those with homozygote recessive variant (TT) were protective [aOR = 0.12 (0.06–0.25)]. Single nucleotide polymorphism of rs3752462 (CT genotype) was associated with increased proteinuria, albuminuria, and reduced eGFR.

**Conclusions:**

We have demonstrated that MYH9 polymorphisms exist among Ghanaian hypertensive patients and rs3752462 polymorphism of MYH9 is associated with CKD. This baseline indicates that further longitudinal and multi-institutional studies in larger cohorts in Ghana are warranted to evaluate MYH9 SNP as an independent predictor of CKD among hypertensive patients in Ghana.

## Background

The antecedents of mortality rate in Africa have shifted in recent years from communicable diseases to a combination of chronic non-communicable diseases (NCDs) and communicable conditions especially, in Ghana [1]. According to the World Health Organisation reports in 2018, 43% of all mortality rates in Ghana are attributed to NCDs [2]. High blood pressure affects entirely 1.13 billion people worldwide and most (approximately 67%) live in low- and middle-income countries [3]. Also, about 6% of people living with hypertension have chronic kidney disease (CKD) and are at risk for progression to end-stage renal disease (ESRD) [4]. Thus, the future risk of NCD forms of CKD, predominantly driven by increased rates of hypertension, is a growing public health concern. In sub-Saharan Africa, an alarmingly 32.3% of hypertensive patients have CKD [5].

CKD has been reported to be predominant among hypertensive patients in the Ghanaian population. For instance, Osafo, Mate-Kole [6] reported a CKD prevalence of 46.9% among hypertensive patients from four polyclinics in Accra, Ghana. Also, among the population from the south-western part of Ghana, CKD prevalence is reportedly 22% among hypertensive patients [7]. Additionally, renal impairment was observed at a rate of 25, 9.5, and 10.5% among hypertensive patients in a District hospital, utilizing Cockcroft Gault (CG), Four-Variable Modification of Diet in Renal Disease (4v-MDRD) and the Chronic Kidney Disease-Epidemiology Collaboration (CKD-EPI) equations, respectively [8]. A more recent multicentre cross-sectional study among Ghanaian hypertensive patients reported a CKD prevalence rate of 26.3% [9].

The introduction of genome-wide association studies (GWAS) has made important contributions to the discovery of genes contributing to hypertensive nephropathy (HN) [10]. Recently, a published study uncovered a new approach to the pathogenesis of CKD among the African American population [11]. While Kao et al. reported a significant association between MYH9 point mutations and the risk of CKD [12], Bostrom and Freedman present a common theme that “risk variants of MYH9 could be a major determinant of excess risk of CKD associated with African ancestry” [13]. The spectrum of MYH9-associated nephropathy is such that MYH9 risk variants exhibit the most remarkable relationship with every common complex kidney disease identified. It also signifies a bias marker on ethnic differences in clinical outcomes [13].

The importance of the MYH9 polymorphisms in the context of CKD is that there is enough evidence in the literature of being a causal variant for CKD in diabetics [14], Lupus Nephritis [15], and non-diabetic population [16, 17]. Aside from the major translational benefits of finding genetic risk variants associated with CKD, they are also likely novel therapeutic targets and also enhance our understanding of disease pathogenesis. No published study has assessed MYH9 polymorphisms and the risk of CKD among hypertensives in the Ghanaian population to be the very best of our knowledge. We, therefore, investigated the transferability of the MYH9-rs3752462 gene polymorphism in terms of its association with CKD among Ghanaian hypertensive patients.

## Methods

### Study design and population

The study was a case-control one conducted between January 2018 and June 2019 at Korle-Bu Teaching Hospital (KBTH) with eligible participants drawn from the hypertension clinic and renal unit. A total of 264 hypertensive patients comprising of 132 case group and 132 control group were included for the study. Hypertensive patients aged 18 years and above without history/signs or clinically diagnosed CKD and Glomerular filtration rate (eGFR) ≥ 60 ml/min/1.73 m2 (CKD-EPI formula) classified as control subjects. Hypertensive patients aged 18-years and above with Glomerular filtration rate (eGFR) < 60 ml/min/1.73 m2 (CKD-EPI formula) or clinically diagnosed CKD. CKD cases of unknown origin were excluded from the study. Cases and control participants with comorbid diabetes and accelerated hypertensive patients were excluded from the study.

### Sample size justification

The sample size for the study was calculated using the Cochran–Armitage trend tests [18]. By considering the additive genetic model at a case: control ratios of 1:1, the rare allele frequency of 0.5, and CKD prevalence of 26.3% among hypertensive patients [9], the sample size needed to achieve the prespecified 0.05 α-level and a power 80 for a two-sided trend test was 264 (132 cases vs 132 controls).

### Anthropometric measurement

An automated sphygmomanometer (OMRON HEM705CP; Omron Matsusaka Co, Matsusaka, Japan) was used for the measurements of blood pressure (BP). Three consecutive readings of blood pressure measurements were taken from the patients’ right arm and the mean of two closest values was recorded. Controlled blood pressure was defined as BP < 140/90 mmHg. Bodyweight of all participants expressed in 0.1-Kg intervals was taken at a fasting state using an automated weighing scale. Portable height rod stadiometers were used for height measurements to the nearest centimetre. BMI was calculated as the ratio of body weight to height (Kg/m^2^).

### Blood sample collection

Five millilitres (5 mL) of the venous blood sample was collected from each participant. Three (3) mL of blood collected into BD vacutainer® gel tubes and 2 mL into a tube containing 2.5 μg of dipotassium ethylenediaminetetraacetic acid (K2 EDTA) as an anticoagulant. Serum preparation was done on the blood samples in the gel tube and stored at − 80 °C until assayed. EDTA samples were processed immediately for gDNA extraction.

### Urinalysis

Early morning urine or spot urine was collected into wide-mouth plastic containers from the respondents and urine protein, albumin, and urine creatinine levels were estimated using a complete chemistry auto-analyser (SELECTRA Pro S) with Ellitec reagents (Namarka, Germany).

### Biochemical assay

Serum samples were analysed for urea, creatinine, fasting blood sugar, total cholesterol, LDL-cholesterol, HDL-cholesterol, and Triglycerides using the SELECTRA Pro S chemistry analyser, (ELITechGroup, France). Dyslipidemia was defined when subjects had either of the following: total cholesterol ≥ 200 or triglyceride 150 mg/dL (1.7 mmol/L) or high-density lipoprotein < 40 mg/dL (1.03 mmol/L) in male < 50 mg/dL (1.29 mmol/L) in females. Hyperglycaemia: Fasting glucose levels ≥6.1 mmol/L.

### Sample processing for DNA analysis

Genomic DNA was extracted from EDTA peripheral blood using the Quick-gDNATM Blood Miniprep Kit (Zymo, Hilden, Germany) following the manufacturer’s protocol and quantified using a NanoDrop 1000 spectrophotometer (Thermo Scientific, Waltham, MA, USA). The DNA samples were amplified using the Tetra primer Amplification Refractory Mutation System (T-ARMS) polymerase chain reaction (PCR).

### Primer design for T-ARMS PCR

The principle of the primer design was based on the use of 2 primer pairs (one pair specific to the area of amplification and the other pair specific to the SNP of interest) to amplify and produce specific band sizes consistent to the particular genotype of the sample being studied all in one single PCR reaction. The primers for this study were designed using PCR Designer for Restriction Analysis of Polymorphism (http://primer1.soton.ac.uk/primer.html) for T-ARMS-PCR as described in [19] The primers used, band sizes and melting temperature is shown in Table 1.

**Table 1:** Primers for rs3752462 genotyping

Primers (5′ - 3′)	Genotype pattern (bp)	Melting Temperature °C
Forward Inner Primer	287 (outer) 199 (T-allele) 140 (C-allele)	69
476 AGGTGTGAGGTCAAAGCAAGCCTTGT 501		
Reverse Inner Primer		
526 CGACCTCATTGAGAAGCCAGTGATTG 501		
Forward Outer Primer		
387 CACCAAAAGGAAGGGGAGTTAAGACACC 414		
Reverse Outer Primer		
673 GTTTGAGCAGCTGTGCATCAATTACACC 646		

### T-ARMS PCR genotyping

**Fig. 1:**
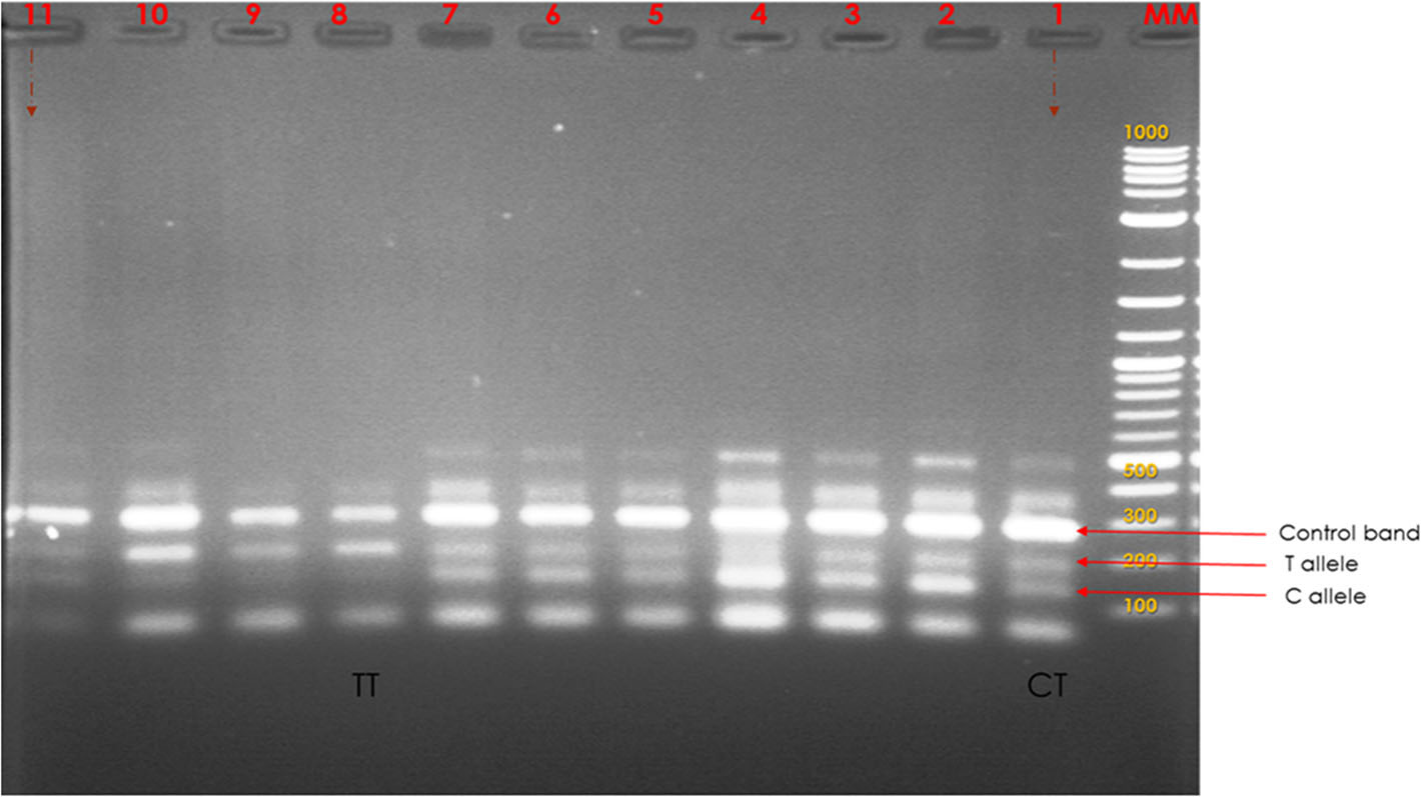
A representative image showing 2% (w/v) agarose gel electrophoresis of T-ARMS Genotyping

PCR reaction was performed in a BIO-RAD PTC-220 thermocycler (Dyad MJ Research) for 45 cycles at an initial denaturation temperature of 95 °C for 5 min, final denaturation at 94 °C for 3 min, annealing at 73 °C for 45 s with decreasing temperature of 0.5 °C per each cycle, initial extension at 72 °C for 3 min and a final extension at 72 °C for 8 min. PCR reactions were carried out in a total volume of 25 μL containing 12.50 μl of Quick-Load 2x Master mix with standard buffer (England Biolabs), 0.5 μM of each of the four primers, 4.0 μM of DNA template and 3.25 uL of sterilized nuclease-free water. Reactions were done twice to evaluate the consistency of the banding patterns for all isolates studied. Products were separated on a 2% (w/v) agarose gel in 1x TAE buffer (40 mM Tris-acetate, 1 mM EDTA, pH 8.0) at 80 V for 2 h (Fig. 1).

### Statistical analysis

All data was entered into Microsoft Excel and double cleaned for multiple entries. Continuous variables were expressed as means ± SD and categorical data were expressed as absolute numbers and proportions. T-test and Wilcoxon signed-rank tests were used to analyze the differences in biochemical and anthropometric parameters between the case and control groups. The Hardy-Weinberg equilibrium analysis of the SNPs was conducted using the Chi-squared test. The genotype and allelic frequencies of SNPs were also assessed by the Chi-squared test. To test for associations between CKD and MYH9-rs3752462SNP, logistic regression models were fitted, where each SNP was presented as a predictor variable whose values were equal to the number of copies of the minor allele (0, 1, 2) in an additive model, or presence of at least one copy of the minor allele (0, 1) in a dominant model or presence of two copies of the minor allele (0, 1) in a recessive model. Sex, age, family history of CKD, BP status, and lipid measurements were included as covariates in the fitted model. The structure of the model was represented as:

Logit [pr (D = 1)] = α + β 1 G + β 2 sex + β 3 age + β 4 family history + β 5 BP status + β 5Lipid measurements.

Where D denotes CKD phenotype; G denotes SNP coded as an additive, dominant or recessive; β denotes the corresponding coefficient for each variable in the model (SNP, sex or age or family history or BP status or BMI or TC or HDL-C or LDL-C), and its exponential was the corresponding odds ratio.

## Results

Table 2 shows the comparison of the basic characteristics between the case and control subjects. The mean age of the control subjects was higher compared to the case subjects (*p* < 0.0001). There were more male subjects than females in the case group whereas more females were found in the control group (*p*-value < 0.0001). A family history of CKD was present in 11.4% of the study case group compared to 0.8% among the control group (*p* < .0001).

**Table 2:** Characteristics of the study participants

Variables	Controls (*n* = 132)	Cases (*n* = 132)	*P*-values
Age (years)^a^	45.9 ± 14.5	61.7 ± 11.7	***< 0.0001***
Gender			
Male	24 (18.2)	77 (58.3)	***< 0.0001***
Female	108 (81.8)	55 (41.7)	***< 0.0001***
Family history of CKD	1 (0.8)	15 (11.4)	***< 0.0001***
Anthropometric indices^a^			
BMI (Kg/m2)	32.2 ± 9.4	35.3 ± 15.8	0.053
SBP (mmHg)	142.4 ± 22.6	167.2 ± 29.5	***< 0.0001***
DBP (mmHg)	87.6 ± 13.4	97.4 ± 21.6	***< 0.0001***
Biochemical analytes			
Total cholesterol (mmol/L)^a^	5.0 ± 1.1	4.5 ± 1.3	***0.001***
Triglycerides (mmol/L)^a^	1.39 ± 0.67	1.45 ± 0.71	0.455
HDL-C (mmol/L)^a^	1.62 ± 0.30	1.40 ± 0.46	< 0.0001
LDL-C (mmol/L)^a^	2.73 ± 0.99	2.45 ± 1.15	0.035
Glucose (mmol/L)^a^	5.6 ± 1.21	5.5 ± 1.3	0.518
Urea (mg/dl)^a^	4.0 ± 1.1	19.0 ± 8.72	***< 0.0001***
Creatinine (mg/dl)^b^	71.0 ± 13.0	889.1 ± 533.76	***< 0.0001***
Urinary protein (mg/dl)^b^	210.0 (120.0–285.0)	530.0 (360.0–760.0)	***< 0.0001***
Urinary Albumin (mg/dl)^b^	10.0 (10.0–20.0)	110.0 (35.0–280.0)	***< 0.0001***
Urinary Creatinine (mg/dl)^b^	151.5 (72.6–223.3)	163.6 (107.4–253.0)	***0.021***
uACR (μg/mg)^b^	92.0 (65.9–172.0)	729.1 (144.3–1783.3)	***< 0.0001***
uPCR (mg/mg)^b^	1.40 (0.90–2.10)	3.05 (1.63–5.30)	***< 0.0001***
Overweight/Obesity	100 (75.8)	125 (94.7)	***< 0.0001***
Uncontrolled BP	47 (35.6)	81 (61.4)	***< 0.0001***
Hyperglycaemia	34 (25.8)	42 (31.8)	0.277
Dyslipidaemia	66 (50.0)	70 (53.0)	0.712
Microalbuminuria (μg/mg)	124 (93.9)	31 (23.5)	***< 0.0001***
Macroalbuminuria (μg/mg)	6 (4.5)	92 (69.7)	***< 0.0001***
rs3752462			
CC	3 (2.3)	3 (2.3)	1.000
CT	42 (31.8)	106 (80.3)	***< 0.0001***
TT	87 (65.9)	23 (17.4)	***< 0.0001***
MAF	0.820	0.576	***< 0.0001***

^a^Variables are presented as mean ± SD and compared using t-test; ^b^represents values presented in median (interquartile ranges) and compared using Mann–Whitney test. Unless otherwise stated, all variables are presented as frequencies and compared using the Chi-square test. Italicized values represent statistically significant values. *BMI* body mass index, *uACR* urine albumin-creatinine ratio, *uPCR* urine protein-to-creatinine ratio

### Metabolic characteristics of the study participants

No significant difference was evident between cases and control subjects in terms of BMI, TG, and fasting glucose levels (*p* > 0.05). There were significant differences between cases and control subjects in all serum analytes and derived ratios (*p* < 0.05) other than those stated above. The systolic and diastolic blood pressure of cases and control subjects differed significantly < 0.005) and were found to be above the recommended cut-off for controlled BP (< 140/90) (Table 2).

Overweight/obesity was high among the case subjects compared to that of the control subjects (94.7% vs 75.8%, *p* < 0.0001). Also, uncontrolled blood pressure was more observed in the case subjects compared with the control subjects (*p* < 0.01). Microalbuminuria was statistically significantly higher in the control group than what was observed for the case group (93.9% vs 23.5%, *p* < 0.0001), whereas clinical albuminuria was more observed among cases than controls (69.7% vs 4.7%, *p* < 0.0001).

Genotype and Allele Frequencies of rs3752462 SNP among Cases and Control Group.

The frequency of the CT genotype of rs3752462 was significantly higher among case subjects compared to control subjects (80.3% vs 31.8%, *p* < 0.000. However, the TT genotype of rs3752462 was more frequent among control participants compared with cases (65.9% vs 17.4%, *p*-value < 0.0001). The frequency of the minor allele (T) was high and equivalent in both groups (97.7%). The genotype frequencies rs3752462 SNPs among the case group deviated from Hardy–Weinberg equilibrium (*p*-value < 0.0001) (Table 2).

Table 3 shows the frequencies, odds ratios, and *p*-values of the rs3752462 genotypes among cases and controls under recessive, dominant, co-dominant, additive, and allele models. As shown in Table 2, age, sex, family history of CKD, blood pressure status and some biochemical analytes were significant effect modifiers. The effect of these modifiers was controlled for in our fitted regression model.

**Table 3:** Frequencies, odds ratios, and *p*-values of rs3752462genevariants among cases and controls under recessive, dominant, co-dominant, additive and allele models

Model	Allele	Controls (*n* = 132)	Cases (*n* = 132)	cOR (95% CI)	aOR (95% CI)
Dominant model	wt/mt CT + mt/mt TT	97.7%	97.7%	1	1
	wt/wt CC	2.3%	2.3%	1.00 (0.20–5.05)	1.10 (0.19–6.29)
Recessive	wt/mt CT + wt/wt CC	34.1%	82.6%	1	1
	mt/mt TT	65.9%	17.4%	0.11 (0.06–0.19)**	0.12 (0.06–0.25)**
Co-dominant model	wt/mt CT + mt/mt TT	68.2%	19.7%	1	1
	wt/mt CT	31.8%	80.3%	8.73 (4.97–15.36)**	7.82 (3.81–16.04)**
Additive model 1	wt/mt CT	93.3%	97.2%	1	1
	wt/wt CC	6.7%	2.8%	0.40 (0.08–2.04)	0.41 (0.07–2.44)
Additive model 2	mt/mt TT	96.7%	88.5%	1	1
	wt/wt CC	3.3%	11.5%	3.78 (0.71–20.0)	4.0 (0.64–24.89)
Allele Model	T-allele	97.7%	97.7%	1	1
	C-allele	34.1%	82.6%	9.16 (5.15–16.30)**	8.29 (4.0–17.27)**

*mt* mutant type, *wt*; ild type, *CI* confidence interval, *cOR* crude odds ratio, *aOR* adjusted odds ratio (adjusted for age, sex, family history of CKD, hypertensive control status, BMI, TC, HDL-C, LDL-C); ** *p*-value < 0.001

The statistical analyses showed that there is a significant difference between the two groups under the recessive and co-dominant models. However, there was no significant difference between the two groups under the dominant and additive models. Under the co-dominant model, rs3752462 was associated with CKD in both the unadjusted [cOR = 8.73 (4.97–15.36)] and adjusted model [aOR = 7.82 (3.81–16.04)]. However, under the recessive model, rs3752462 was protective of CKD in both the unadjusted and adjusted model (*p*-value < 0.001).

**Fig. 2:**
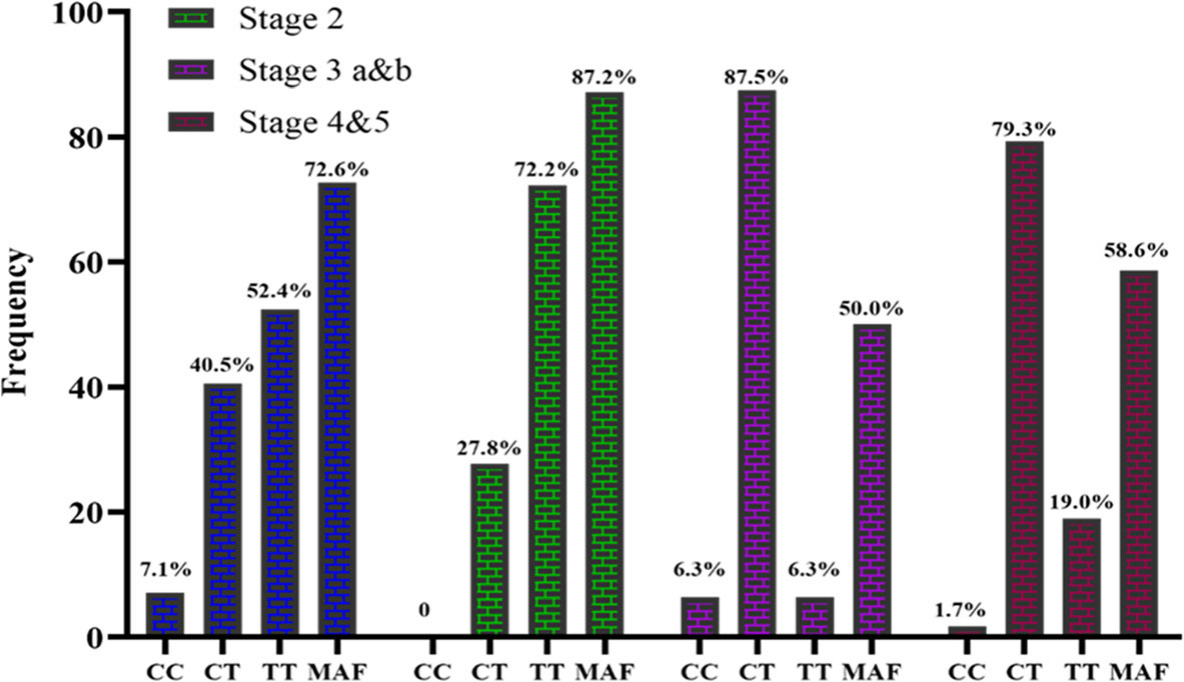
A representative image of the distribution of rs3752462 gene variants stratified by CKD stages. MAF- minor allele frequency

We compared the distribution of rs3752462 gene variants by CKD stages. Due to small numbers, stage 3A &B, as well as Stage 4&5, were combined. The proportion of TT variants was higher (> 50%) among hypertensive patients in Stage 1 and 2 of CKD. Also, the percentage of the CT genotype was higher (> 60%) among patients at the extreme stages. The minor allele frequencies (T) were 72.6 and 87.2%, among patients in Stage 1, 2, respectively. Also, among patients grouped as 3a&b and 4&5, the minor allele frequencies were respectively, 50.0 and 58.6% (Fig. 2).

We compared the features of renal abnormalities among patients with CT and TT genotype and the results presented in Table 4. The levels of serum creatinine, urea, uPCR, and uACR, were high among participants with the CT genotype compared with those with the TT genotype (*p*-value < 0.01). Urine creatinine levels did not differ between the two groups (Table 4).

**Table 4:** Comparison of features of renal abnormalities by genotypes of rs3752462

Variable	CT genotype *n* = 148	TT genotype *n* = 110	*P*-value
Urea (mmol/l)	12.4 (5.4–22.3)	4.20 (3.3–5.9)	< 0.0001
Urine protein (mg/dl)	450.0 (235.0–710.0)	270.0 (170.0–370.0)	< 0.0001
Urine Albumin (mg/dl)	50.0 (10.0–210.0)	10.0 (10.0–30.0)	< 0.0001
uPCR (mg/mg)	2.70 (1.43–9.40)	1.60 (1.00–2.30)	< 0.0001
uACR (μg/mg)	395.3 (122.2–1376.9)	92.0 (67.3–175.4)	< 0.0001
Serum Creatinine (mg/dl)	538.9 (84.5–1073.2)	75.8 (66.6–94.1)	< 0.0001
Urine Creatinine (mg/dl)	140.1 (94.4–237.6)	169.1 (115.3–245.4)	0.290

*uACR* urine albumin-creatinine ratio, *uPCR* urine protein-to-creatinine ratio. All values are presented as median (interquartile ranges) and compared using the Mann Whitney Test. Italized values represent statistically significant values

## Discussion

The future risk of NCD forms of CKD, predominantly driven by increased rates of hypertension, is a growing public health concern in Ghana [6–9], and in some African countries, it is a death sentence [20]. Although the epidemiology of CKD in Africa is well elucidated to some extent [21], current evidence has uncovered new approaches to understanding the pathogenesis of CKD [11]. MYH9- rs3752462 polymorphism by far has produced an impressive association with CKD among diabetics [14], Lupus Nephritis [15], and non-diabetic population [16, 17]. However, there is an extreme limitation of data on SNP of MYH9-rs3752462 associated CKD in the Ghana population. We, therefore, provide baseline evidence of MYH9-rs3752462 gene polymorphism among Ghanaian hypertensive patients and evaluated its association with CKD.

Our findings indicated that there is an excess of the minor allele (MAF) of rs3752462 among the case and control subjects (57.6% vs. 82.0%). Likewise, our results also revealed that heterozygote genotype (CT) of rs3752462 was predominant among case subjects while the homozygote recessive (TT) genotype of rs3752462 was more frequent among control subjects. The observed frequencies are higher compared to the reports from a study by Oleksyk et al. who noted that Africans have the lowest heterozygosity for rs3752462 (23.2%) compared to the Americans (42.4%), South Central Asians (51.9%), East Asians (38.1%), Europeans (41.4%) and Middle East (43.8%) population, based on evidence of historical selection in Africa [22].

Oleksyk et al. [22] further described that this observation among the African population may be a consequence of natural selection but should only be interpreted in conjunction with other indicators of a selective sweep. Furthermore, the population frequencies of SNP rs3752462 from 1000 genomes were 4.4% for the homozygote dominant (CC), 36.3% for the heterozygote (CT), and 59.3% for the homozygote recessive (TT) respectively. Nevertheless, the minor allele from 1000 genomes has the highest frequencies in Africa (78.6%) and Middle East (79.3%) [23], which is similar to our observation among Ghanaian hypertensive patients.

Familial aggregation of end-stage renal disease has been demonstrated to be the strongest in the African-American population [15, 24]. It becomes apparent that CKD susceptibility from systemic disorders could have an inherited basis [13] and understanding of the distribution of risk genes among a population serves an important tool for genetic studies. Similar to other related studies [17, 25], we observed that MYH9 polymorphic variant rs3752462 is closely associated with CKD among hypertensive patients in Ghana. Patients with the heterozygote (CT) genotype of rs3752462 were associated with 8-times increased likelihood of developing CKD even when covariates and other related risk factors were controlled. However, patients with the homozygote recessive genotype (TT) were likely protective of CKD. These findings are consistent with the findings of Tavira et al. [17] who indicated that SNP rs3752462 is an independent predictor of reduced eGFR in the Spanish RENASTUR population. Hence, SNP rs3752462 may be associated with CKD in Ghanaian hypertensive patients. For the reason that CKD is often silent until late stages, genotyping of MYH9-rs3752462 among Ghanaian hypertensive patients could help identify susceptible patients for prevention.

A study by Tavira et al. [17] amplified the transcripts from heart tissues of patients who are heterozygotes for rs3752462, with primers that matched exons 12 and 14 and found a single PCR band that corresponded to a normal MYH9 sequence. The authors concluded that the lack of aberrant transcripts suggested a lack of effect on pre-mRNA splicing, which appears that rs3752462 enhances CKD susceptibility by regulatory effect rather than functional effect. Besides, the effect of rs3752462 and for that matter, MYH9 gene on CKD has been attributed to linkage disequilibrium with functional SNP variants in other related studies, where APOL1 variants have become the major theme for discussion [16, 26]. In this study, individuals with the heterozygote variant of rs3752462 had significantly lower eGFR compared to those who are homozygote recessive. Additionally, majority of patients with CKD stages 3, 4, or 5 had the CT genotype of rs3752462. This observation has been demonstrated in a previous study [17], where patients with the minor allele of rs3752462 were more likely to have reduced eGFR < 60 ml/ min./1.73m^2^ after adjusting for the effect of age, gender, and lipid profile.

We also observed that patients with the CT genotype of SNP rs3752462 of the MYH9 gene showed characteristics of high uACR, uPCR, and high urine albumin and protein excretion levels. One key feature of MYH9 gene mutation in the podocyte and mesangial cells is that it disrupts the cytoskeleton and causes membrane instability [27]. Thus, abnormalities of the MYH9 gene may explain the presence of proteinuria and the evolution of CKD in patients affected [28, 29]. It has been shown that 30.0% of all patients who have the MYH9 gene mutation exhibit renal derangements [30], characteristics of early-onset proteinuria, and rapid progression to CKD [29]. More so, nephropathy associated with mutation of the MYH9 gene usually progresses to terminal CKD around the age of 30 years, yet cases of more advanced age have been described [31]. Thus, screening for MYH9 gene mutations in the Ghanaian population could enhance the detection of CKD susceptible cases. Although the rs3752462 T allele (CT + TT genotypes) were statistically significant and our sample sizes were enough to reach a power 80, our work was based on a limited number of individuals from a single Ghanaian population.

Collectively, our findings are consistent with reports from other studies, however, we should mention here that the study had some limitations. The study has a small sample size and cases in this present study were defined as hypertensive patients with CKD and no specialized test or renal biopsies were performed to exclude other secondary causes other than diabetes and HIV. Subjects with primary glomerular disease or CKD of unknown etiology (CKDu) were excluded even though renal damage of hypertension diagnosed by clinicians may often not be related to systemic hypertension.

## Conclusions

We have demonstrated that MYH9 polymorphisms exist among Ghanaian hypertensive patients and rs3752462 polymorphism of MYH9 is associated with CKD. This baseline indicates that further longitudinal and multi-institutional studies in larger cohorts in Ghana are warranted to evaluate MYH9 SNP as an independent predictor of CKD among hypertensive patients in Ghana. Screening for MYH9- rs3752462 risk variants may eventually be useful in the Ghanaian population, especially high-risk populations such as hypertensives with or without a family history of CKD.

## Data Availability

The datasets used and/or analysed during the current study are available from the corresponding author on reasonable request.

## Abbreviations

MYH9: Non-muscle myosin heavy chain 9 gene
CKD: Chronic kidney disease (CKD)
T-ARMS: Tetra primer Amplification Refractory Mutation System
PCR: Polymerase chain reaction
